# *Cymbopogon winterianus* Essential Oil Attenuates Bleomycin-Induced Pulmonary Fibrosis in a Murine Model

**DOI:** 10.3390/pharmaceutics13050679

**Published:** 2021-05-09

**Authors:** Lívia A. Tavares, Allan A. Rezende, Jymmys L. Santos, Charles S. Estevam, Ana M. O. Silva, Jaderson K. Schneider, John L. S. Cunha, Daniela Droppa-Almeida, Ivan J. Correia-Neto, Juliana C. Cardoso, Patricia Severino, Eliana B. Souto, Ricardo L. C. de Albuquerque-Júnior

**Affiliations:** 1 Post-Graduating Program in Biotechnology, Brazilian Biotechnology Northeast Network, Tiradentes University, Aracaju 49010-390, Brazil; livianjos15@hotmail.com (L.A.T.); allan_a.rezende@hotmail.com (A.A.R.); juaracaju@yahoo.com.br (J.C.C.); pattypharma@gmail.com (P.S.); 2 Post-Graduating Program in Biotechnology, Brazilian Biotechnology Northeast Network, Federal University of Sergipe, São Cristóvão 49100-000, Brazil; jymmyslopes@yahoo.com.br (J.L.S.); cse.ufs@gmail.com (C.S.E.); 3 Department of Nutrition, Federal University of Sergipe, São Cristóvão 49100-000, Brazil; anamaraufs@gmail.com; 4 Post-Graduating Program in Industrial Biotechnology, Tiradentes University, Aracaju 49010-390, Brazil; jadersonqmc@gmail.com; 5 Post-Graduating Program in Stomatopathology, University of Campinas, Campinas 13083-970, Brazil; lennonrrr@gmail.com; 6 Post-Graduating Program in Health and Environment, Tiradentes University, Aracaju 49032-490, Brazil; danieladroppa@gmail.com; 7 Post-Graduating Program in Dental Science, University of São Paulo, São Paulo 05508-010, Brazil; ivanc.neto@gmail.com; 8 Faculty of Pharmacy, University of Coimbra, Pólo das Ciências da Saúde, Azinhaga de Santa Comba, 3000-548 Coimbra, Portugal; 9 CEB—Centre of Biological Engineering, University of Minho, Campus de Gualtar, 4710-057 Braga, Portugal

**Keywords:** pulmonary fibrosis, terpenes, Wistar rats, myofibroblasts, histological labelling, immunohistochemistry

## Abstract

The essential oil of *Cymbopogon winterianus* (EOCW) is a natural product with antioxidant, anti-inflammatory, and antifibrotic properties. We studied the effect of EOCW in the progression of histological changes of pulmonary fibrosis (PF) in a rodent model. The oil was obtained by hydrodistillation and characterized using gas chromatography–mass spectrometry. Intratracheal instillation of bleomycin was performed in 30 rats to induce PF, while Sham animals were subjected to instillation of saline solution. The treatment was performed using daily oral administration of distilled water, EOCW at 50, 100, and 200 mg/kg, and deflazacort (DFC). After 28 days, hemogram and bronchoalveolar lavage fluid (BALF), tissue levels of malondialdehyde (MDA), superoxide dismutase (SOD), and catalase (CAT) were assayed. Histological grading of PF, immunohistochemical expression of α-smooth muscle actin (α-SMA), and transforming growth factor-β (TGF-β) were also analyzed. The EOCW major compounds were found to be citronellal, geraniol, and citronellol. EOCW significantly reduced inflammation in BALF, reduced MDA levels, and increased SOD activity. EOCW attenuated histological grading of PF and reduced immunohistochemical expression of α-SMA and TGF-β in a dose-dependent way, likely due to the reduction of oxidative stress, inflammation, and TGF-β-induced myofibroblast differentiation.

## 1. Introduction

Idiopathic pulmonary fibrosis (PF) is a chronic interstitial lung disease that affects over 3 million individuals worldwide, with a mean survival time of about 3 years, characterized by progressive deposition of fibrotic tissue in the lungs and overall poor prognosis [1,2]. Studies have suggested that the incidence and prevalence of idiopathic PF is continuously growing, and that PF is strongly associated with advanced age [3,4].

The main pathologic changes that play a critical role in the pathogenesis of idiopathic PF are oxidative stress, inflammation, and collagen deposition, but the comprehensive understanding of the pathobiology of the disease remains elusive [5]. However, micro-injury of the alveolar epithelium induced by environmental and microbial exposures (metal and wood dust, viruses, and drugs) and individual genetic factors (dysfunctions of the MUC5B gene, causing reduction of mucociliary clearance enhancing lung injury) have been recognized as the first drivers of idiopathic PF [2,6]. Subsequently, fibroblast and myofibroblasts collections that actively produce extracellular matrix, including high levels of collagen, are hallmarks of idiopathic PF progression. The source of these cells has been related to epithelial–mesenchymal transition (EMT), a pathophysiological process that is defined by the detection of several biomarkers that mirror the loss of epithelial phenotype and the gain of mesenchymal one, such as cytoskeletal proteins involved in cell contraction (e.g., α-smooth muscle actin) [7]. EMT events are involved in the direct conversion of damaged lung epithelial cells into mesenchymal fibroblasts, and the persistence of EMT-inducing signals seems to promote extracellular matrix accumulation causing tissue scarring in idiopathic PF [8,9].

Transforming growth factor beta (TGF-β) is a cytokine that plays a key role in the pathogenesis of idiopathic PF [10]. Increased expression of TGF-β has been demonstrated to precede collagen synthesis and deposition in animal models of lung fibrosis [11], and adenoviral-mediated gene transfer of active TGF-β has induced severe fibrosis in rodent lungs [12]. TGF-β modulates EMT-related transdifferentiation of pulmonary fibroblasts and type I alveolar cells into myofibroblasts, such as plasma membrane junctional and adhesion complexes adaptation, and cytoskeleton reorganization [13]. An increased number of myofibroblasts, identified by their expression of α-smooth muscle actin (α-SMA), has been related to the progression of pulmonary fibrosis [14].

The multifactorial etiopathogenetic factors involving continuous oxidative stress, unresolved inflammation, and tissue scarring have made the management of the idiopathic PF so challenging that few therapeutics have succeeded in the clinic, and they have failed to improve patient survival [15]. As most therapeutic agents (corticosteroids, antifibrotic, and immunosuppressant drugs) have focused on one step at a time of idiopathic PF pathogenesis, the results tend to be unpromising [16,17]. Thus, therapeutic approaches based on traditional knowledge, such as plant-derived compounds, able to act in different steps of idiopathic PF pathogenesis, might be a successful strategy to treat the disease [18].

*Cymbopogon winterianus* Jowitt is an aromatic grass cultivated in India and Brazil. The essential oil obtained from its leaves has demonstrated anti-inflammatory and antioxidant properties [19] and low toxicity [20]. These biological properties are potentially attributed to its the major monoterpenes, namely, geraniol (40.06%), citronellal (27.44%), and citronellol (10.45%) [21]. In fact, geraniol reduces pro-oxidative lipid peroxidation and nitric oxide (NO) and reactive oxygen species (ROS) production [22], whereas citronellal inhibits pro-inflammatory 5-lipoxygenase (5-LOX) synthesis [23]. Citronellol inhibits inducible nitric oxide synthase (iNOS) and cyclooxygenase-2 (COX-2) expression, resulting in antioxidant and anti-inflammatory effects [24].

There is also evidence that the EOCW chemical constituents modulate the fibrosis process in different tissues. Geraniol attenuates fibrosis, prevents atherogenesis, and exerts anti-inflammatory effects by the NF-κB signaling pathway on atherogenic diet induced fibrosis in hamsters [25]. Furthermore, the essential oil of *Cichorium glandulosum* Boiss et Huet, whose major chemical constituent is citronellol (74.060%), reduces the extracellular fibrillar proteins deposition, such as collagen and elastic fibers, on carbon tetrachloride-induced liver fibrosis in rats [26].

Considering the antioxidant, anti-inflammatory, and antifibrotic properties of the essential oil of *Cymbopogon winterianus*, and its chemical composition, we hypothesize that it may prevent or attenuate the progression of histological changes of pulmonary fibrosis in a bleomycin-induced murine model.

## 2. Materials and Methods

### 2.1. Sampling Site and Plant Material

Fresh leaves of *Cymbopogon Winterianus* were obtained from the Brazilian Agricultural Research Corporation (EMBRAPA), who collected the samples in July of 2018 in Aracaju (coordinates: 10°57′02.4” S 37°03′07.4” W), Sergipe, Brazil. The specimens were deposited in the herbarium of the Department of Botany nf the Tiradentes University with the registration no. 0844. 

### 2.2. Extraction and Analysis of the Essential Oil

The fresh leaves of *Cymbopogon Winterianus* were dried at 60 ± 1 °C for four days in a drying oven (MA035/5, Marconi^®^, Piracicaba, São Paulo,, Brazil). Hydrodistillation was used to obtain the essential oil of the leaves in a Clevenger-type apparatus. The obtained essential oil was separated from the aqueous phase and stored in an amber bottle in a freezer (at −4 °C) until further used. The chemical analysis of its components was performed by a GC/MS (GCMSQP2010 Ultra, Shimadzu Corporation, Kyoto, Japan) equipped with an AOC-20i autoinjector (Shimadzu Corporation, Kyoto, Japan), following a previously described method [27], with some modifications. The separations were performed on 30 m, Rtx^®^-5MS Restek fused silica capillary column (5% diphenyl-95% dimethylpolysiloxane) with a 0.25 mm internal diameter and 0.25 mm film thickness. Helium 5.0 was used as the carrier gas at a flow rate of 1.0 mL/min. The injection temperature was 280 °C. The volume of 1.0 μL (10 mg/mL) of sample was injected at a split ratio of 1:30. The oven temperature was programmed isothermally at 50 °C for 1.5 min, followed by a rate increase of 4 °C/min until reaching 200 °C, and then at 10 °C/min up to 300 °C, which was kept for 5 min. For the GC/MS, the ionic capture detector impact energy was 70 eV. The fragments were analyzed by a quadrupolar system programmed to filter fragments/ions with m/z from 40 to 500 Da and detected by an electron multiplier. The data were processed with the aid of GCMS Postrun Analysis software (Labsolutions, Shimadzu Corporation, Kyoto, Japan). The components were identified by a comparison of their retention times with those available in the literature [28]. The retention index was determined using the Van den Dool and Kratz (1963) equation [29], for a homologous series of n-alkanes (nC9–nC18). The components of the essential oil were also identified by comparing their mass spectra with the spectra available in the WILEY8, NIST107, and NIST21 equipment databases, which allow the comparison of mass spectral data sets and the use of a minimum similarity index of 80%.

### 2.3. Experimental Procedures of Lung Fibrosis: Induction and Treatment

The animal experiments were approved by the Ethics Committee on Animal Research of the Tiradentes University (CEUA/UNIT) through Opinion No. #020917. Ethics principles Use of Laboratory Animals of the Brazilian Society of Laboratory Animal Science (SBCAL/COBEA) were followed together with the 3R principles of the EU Directive 2010/63/EU transferred to the national Decreto-Lei 113/2013 (in Portugal), the 2001/83/EC and 86/609/EEC (on the protection of animals used for experimental and other scientific), and the Amsterdam protocol on animal protection and welfare of 1997 FP7 Decision number 1982/2006EC. Thirty-six adult male Wistar rats (*Rattus norvegicus albinus*) weighing 225 ± 25 g were randomly assigned into six experimental groups (Table 1). Animals were housed in plastic cages with bedding of wood shavings, which was replaced daily, under controlled temperature at 22 °C and a 12 h light/dark regimen, with water and food ad libitum (Labina^®^; Purina, São Paulo, Brazil). Bleomycin-induced lung fibrosis was performed according to Bahri et al. (2017) [5]. The animals were subjected to dissociative anesthesia with intraperitoneal administration of 0.10 mL/100 g of 10% ketamine (Ketamine^®^, Rhobifarma Indústria Farmacêutica Ltda, Hortolândia, São Paulo, Brazil) and 0.25 mL/100 mg of xylazine (Anasedan^®^ - Sespo Ind e Com. Ltda, Paulínia, São Paulo, Brazil, ). Briefly, induction of fibrosis was carried out by intratracheal instillation of 5.0 mg/kg body weight (bw) of bleomycin sulfate (Cinaleo^®^, Laboratories Meizler, São Paulo, Brazil) dissolved in 0.5 mL of sterile saline. The same procedures were performed in the control group (Sham), using saline solution instead of bleomycin. The treatment of the animals proposed for the different experimental groups started three days after these procedures. Deflazacort (Calcort^®^, Merrell Lepetit, São Paulo, Brasil), soybean oil, and EOCW were administered daily by gavage, for 28 days. The animals were euthanized by cardiac puncture exsanguination, and the lungs were surgically removed. The left lung was fixed in 10% phosphate-buffered formalin (pH 7.4) for further histological procedures. The right accessory pulmonary lobes were frozen (−80 °C) for further colorimetric assays.

### 2.4. Assessment of the Body Weight of the Animals

The body weight of the animals was assessed at the beginning and at the end of the experiment. The percentage of body weight gain in each group was calculated using the Equation (1).

(1) $$BWg=\frac{fBw-iBw}{fBw}\times 100$$

where *BWg* is the percentage of body weight gain, *fBW* is the body weight at the end of the experimental time, and *iBW* is the body weight at the beginning of the experimental time. 

### 2.5. Hematological Analysis of the Peripheral Blood

Hematologic analysis was performed using the automatic hematologic analyzer Sysmex Xs1000i (Sysmex America, Inc., Mundelein, IL, USA). Differential leukocyte counting was performed with an optical microscopy after staining with Pappenheim’s method for each case. Data were expressed as absolute number of cells per mm^3^ of peripheral blood.

### 2.6. Analysis of the Bronchoalveolar Lavage Fluid (BALF)

After cardiac puncture exsanguination, 1 mL of sodium phosphate buffer was slowly injected into the lungs through a catheter and then immediately aspirated. Samples were centrifuged, supernatant was stored at –80 °C, and the pellet was resuspended in 250 μL of sodium phosphate buffer. The total number of cells was assessed by direct counting in a hemocytometer using the Trypan blue exclusion method. For cell differentiation analysis, 200 μL of cell suspension was added in a cytospin slide chamber (Shandon EZ Double Cytofunnel, Thermo Scientific, Waltham, MA, USA), spun at 800 rpm for 5 min in a Cytospin 4 (Thermo Scientific, USA) and stained with Stain Set Diff-Quik (Siemens Healthcare Diagnosis Inc., Newark, NJ, USA). Percentages of macrophages, neutrophils, and lymphocytes were obtained and adjusted by total cell number.

### 2.7. Assessment of Lipid Peroxidation and Mda Tissue Levels

Lipid peroxidation was determined based on the detection of thiobarbituric acid (TBA) reactive products [17]. A reaction with TBA can detect small amounts of lipid peroxides, and more particularly, the free malondialdehyde (MDA) produced during the oxidative breakdown of lipids and polyunsaturated fatty acids. Briefly, we incubated both lung supernatant and sodium phosphate buffer at 37 °C for 1 h, and the mixture was centrifuged after being precipitated with 10% TCA (trichloroacetic acid). Then, 1% TBA was added to the supernatant, and the mixture was placed in the boiling water for 15 min. The absorbance was read at 532 nm and expressed in nmol/mg protein using a molar extinction coefficient of 156,000 M/cm. The determination of total proteins was carried out using the methodology established by the Labtest^®^ brand commercial kit.

### 2.8. Assessment of Superoxide Dismutase (SOD) Tissue Activity

The organs that were removed were washed thrice in potassium chloride solution (1.15% KCl), and then homogenized (1:5 *w*/*v*) with a solution containing KCl, phenylmethylsulfonylfluoride (PMSF 100 m/mol), and Triton solution (10%). Homogenates were centrifuged at 3000× *g* for 10 min at 4 °C, and the supernatant was stored at −80 °C for the determination of oxidative stress markers (TBARS and SH). Superoxide dismutase (SOD) activity was based on the inhibition of the auto-oxidation of epinephrine to adenochrome in the presence of SOD (pH 10.2) [17]. Briefly, 20 mL of epinephrine (5 mg/mL) was added to the assay mixture containing 10 mL of bovine catalase (0.4 U/mL) and 62.5 mM sodium carbonate–sodium bicarbonate buffer. One unit of SOD is defined as the enzyme required to inhibit the quantity of adenochrome generated by 50%. The absorbance was recorded at 480 nm.

### 2.9. Assessment of Catalase (CAT) Tissue Activity

In the samples prepared as previously described, CAT activity was assessed using the method previously reported by Bahri et al. (2020) [17] at 240 nm. Briefly, the reaction mixture included H_2_O_2_ in 0.019 M, 0.05 M phosphate buffer (pH 7), and 0.03 mL of lung sample. CAT activity was expressed as µmole of H_2_O_2_ consumed/min/mg of protein.

### 2.10. Histological Procedures and Assessment of the Histological Grading of Bleomycin-Induced Lung Fibrosis

For histological analysis, the formalin-fixed lung samples were dehydrated in ethanol, diaphanized in xylene, and embedded in paraffin. Then, 20 serial histological sections were obtained from the lung sample. Four of them were stained in hematoxylin/eosin (HE) and six in Masson’s trichrome (5.0 μm thick). The other 10 histological sections (3.0 μm thick) were used for the immunohistochemical study. All of the histological analysis was blindly conducted by two examiners previously calibrated. In order to assess the severity and the extension of lung fibrosis, a semi-quantitative grading system described by Ashcroft et al. (1988) [30] and modified by Hubner et al. (2008) [31] was used. Briefly, 10 histological fields (400×, 0.025 mm^2^) of each section (five from the upper and five from the lower halves of the left lungs) were analyzed and classified as described in Table 2. Data were expressed as median and interquartile intervals.

### 2.11. Analysis of the Immunohistochemical Expression of α-SMA and TGF-β in the Lung Tissue

Both of the immunohistochemical reactions were performed on five 3.0-µm-thick sections of paraffin-embedded tissues. Antigen retrieval was performed with citrate buffer solution (pH 6.0) for 15 min in an electric pressure cooker. Endogenous peroxidase activity was suppressed with 10% H_2_O_2_, in five cycles of 5 min each. Subsequently, the sections were incubated with primary antibodies for 2 h. Primary antibodies included: anti-α-SMA (clone h-CD, dilution 1:400; Dako, Glostrup, Denmark) and anti-TGF-β (clone 17, 1:50 dilution; Novocastra Laboratories, Newcastle upon Tyne, UK). Immunohistochemical staining was performed with Advance (Dako, Hamburg, Germany), following the manufacturer’s instructions. Slides were then exposed to diaminobenzidine tetrahydrochloride (DAB; Sigma-Aldrich, St Louis, MO, USA) and counterstained with Carazzi’s hematoxylin. Twenty histological fields (800×, 0.0625 mm^2^), 10 from the upper and 10 from the lower halves of the lung histological sections, were randomly selected and recorded, and positive cells (identified by cytoplasmic brown stain) were counted. Data were expressed as mean number of positive cells/histological field (0.0625 mm^2^).

### 2.12. Statistical Analysis

Statistical analysis was performed in Graph Pad Prism software, version 7.0 (GraphPad Software, San Diego, CA, USA). Analysis of the normality of distribution of data was performed using the Shapiro–Wilk test. Gaussian data (expressed as mean ± standard mean error) were analyzed using analysis of variance (ANOVA) and Tukey’s multiple comparisons test. Non-gaussian data (expressed as median and interquartile amplitude) were analyzed using the Kruskal–Wallis test and Dunn’s multiple comparisons test. The significance level adopted for all of the tests was 5% (*p* < 0.05).

## 3. Results

The essential oil of *Cymbopogon winterianus* presented as a colorless oil, with a distinct odor and a yield of 1.14%. The identification of the components in the sample is described in Table 3 and included 47 compounds. Among them, 17 oxygenated monoterpenes, 13 oxygenated sesquiterpenes, 7 sesquiterpenes, 5 monoterpenes, 2 aldehydes, 2 ketones, and 1 acid. Citronellal (32.61%), geraniol (22.83%), and citronellol (14.37%) were the main oxygenated monoterpenes found.

Table 4 shows the animals’ body weight on the first and on day 28. Only the group EOCW200 showed a gain in body weight over the experimental period, although this was significantly lower than observed in the Sham group (*p* < 0.001). On the other hand, the Vehicle group and all of the other treated groups (EOCW50, EOCW100 and DFC) showed statistically similar percentages of body weight loss (*p* > 0.05). 

Table 5 shows the assessment of hematological parameters in the groups at the end of the experimental time. Although significant differences were observed in some parameters of both red and white cells of the peripheral blood, all of the values remained within the normal range of the reference. These findings suggest that no relevant status of hematological change was observed under the experimental conditions in the bleomycin-induced pulmonary fibrosis model.

**Figure 1:**
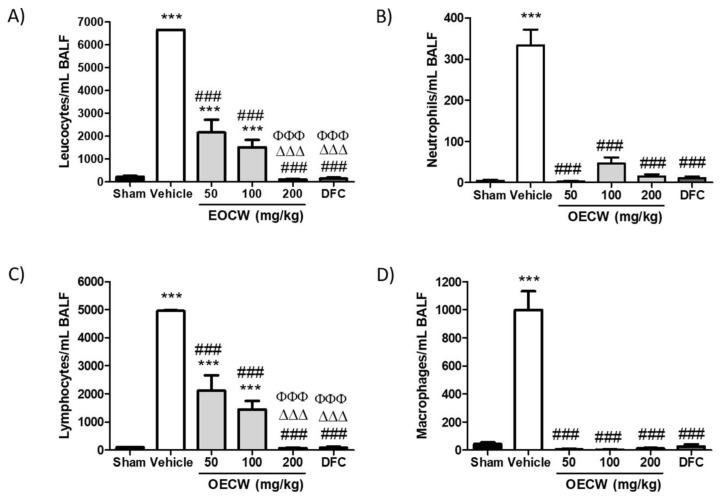
Values obtained from bronchoalveolar lavage cells: (**A**) total cells; (**B**) neutrophils; (**C**) lymphocytes, and (**D**) macrophages. Significant differences compared to the Sham group are expressed with *** *p* < 0.001; significant differences compared to the Vehicle group are expressed as ### *p* < 0.001; significant differences compared to the OECW 50 group are expressed as ΔΔΔ *p* < 0.001; significant differences compared to the OECW 100 group are expressed as ΦΦΦ *p* < 0.001. (Kruska–Wallis, followed by Dunn’s multiple comparisons test).

As demonstrated in Figure 1, a significant increase in the BALF total leukocytes counts was observed in group Vehicle in comparison with Sham (*p* < 0.001). Although a significant decrease in the leukocytes counts was determined by the administration of EOCW at 50 and 100 mg/kg (*p* < 0.001), the leukocyte counts remained significantly above the Sham group (*p* < 0.001). However, the treatment with EOCW at 200 mg/kg and deflazacort not only promoted a significant decrease in the BALF leukocyte counts in comparison with groups Vehicle, EOCW50, and EOCW100 (*p* < 0.001), but also reduced them to values statistically comparable to the Sham group (*p* > 0.05). Differential analysis showed increase in neutrophils, lymphocytes, and macrophages counts in Vehicle (*p* < 0.001). All of the treatment protocols tested in this study similarly promoted significant reduction of neutrophils and macrophages to the basal ranges observed in Sham (*p* < 0.001), and there was no significant difference between them (*p* > 0.05). Regarding lymphocytes, however, although EOCW at 50 and 100 mg/kg determined a significant decrease in the cell counts (*p* < 0.001), only the administration of EOCW at 200 mg/kg and deflazacort promoted a reduction intense enough to bring the values to the basal ranges seen in Sham (*p* > 0.05).

**Figure 2:**
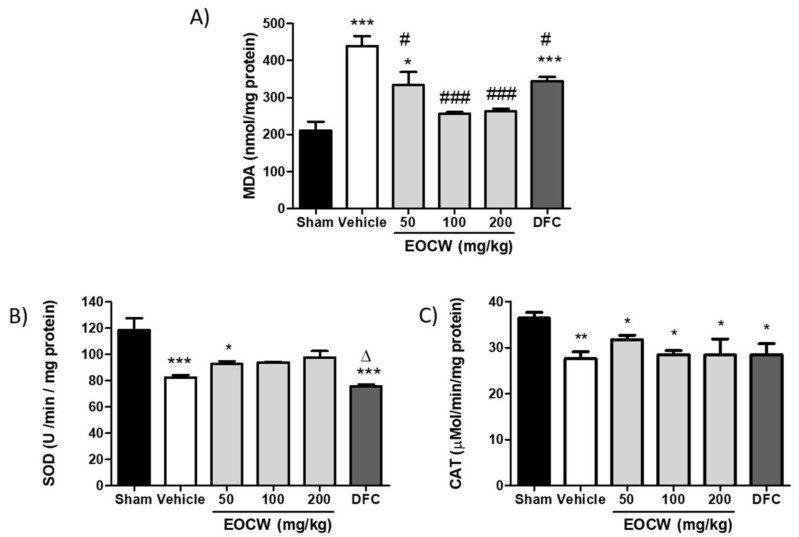
Effect of EOCW on oxidative stress parameters in bleomycin-induced lung fibrosis: (**A**) MDA production, (**B**) SOD activity, and (**C**) CAT activity. Significant differences compared to the group Sham are expressed as * *p* < 0.05, ** *p* < 0.01 and *** *p* < 0.001; significant differences compared to the Vehicle group are expressed as # *p* < 0.05 and ### *p* < 0.001; significant differences compared to the EOCW50 group are expressed as Δ *p* < 0.05.

The tissue contents of MDA were assessed by the thiobarbituric acid reactive substance test (TBARs) in samples of lung tissue from animals with bleomycin-induced pulmonary fibrosis (Figure 2A). Increased levels of MDA were observed in group Vehicle (439.8 ± 26.7 nmol/mg) in comparison with group Sham (211.5 ± 23.1 nmol/mg; *p* < 0.001). Significant reduction of MDA tissue levels in comparison with group Vehicle was observed in groups EOCW 50 (333.8 ± 35.1 nmol/mg; *p* < 0.05), EOCW100 (256.6 ± 4.1 nmol/mg; *p* < 0.001), EOCW200 (262.6 ± 7.2 nmol/mg; *p* < 0.001), and DFC (344.8 ± 10.8 nmol/mg; *p* < 0.05). In addition, the MDA levels observed in groups EOCW100 and EOCW200 were statistically comparable to group Sham (*p* > 0.05). A significant decrease in SOD activity was observed in Vehicle (82.3 ± 2.0 U/min/mg; *p* < 0.001), EOCW50 (92.8 ± 1.9 U/min/mg; *p* < 0.05), and DFC (82.3 ± 2.0 U/min/mg; *p* < 0.001) groups in comparison with that of Sham (75.5 ± 1.5 U/min/mg). However, the SOD activity in groups EOCW100 (94.8 ± 0.4 U/min/mg) and EOCW200 (97.7 ± 4.8 U/min/mg) was statistically comparable to Sham (*p* > 0.05) (Figure 2B). Although no significant difference was observed between the values of CAT activity obtained in groups Vehicle (27.5 ± 1.5 µMol/min/mg), EOCW50 (31.8 ± 0.9 µMol/min/mg), EOCW100 (28.5 ± 0.8 µMol/min/mg), EOCW200 (28.4 ± 3.5 µMol/min/mg), and DFC (28.5 ± 2.3 µMol/min/mg) (*p* > 0.05), they all significantly decreased in comparison with the Sham group (36.4 ± 1.2 µMol/min/mg) (*p* < 0.05; Figure 2C).

**Figure 3:**
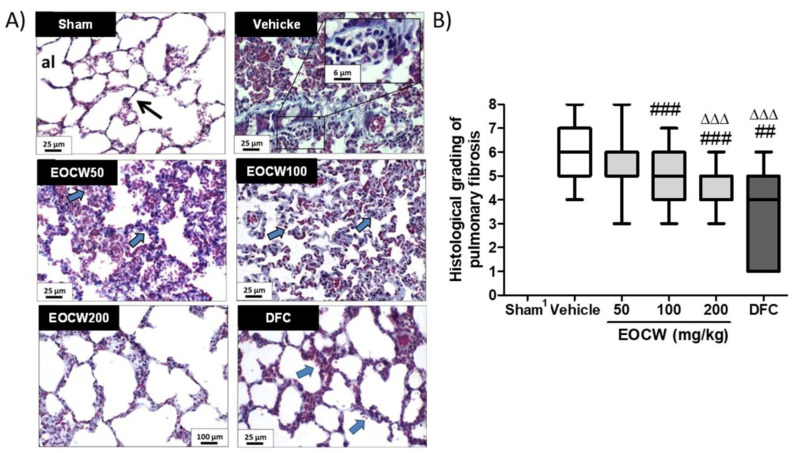
(**A**) Histological features observed in the pulmonary tissue of the experimental groups. Typical regular alveolar spaces (al), with thin septa, are seen in group Sham, whereas groups Vehicle and EOCW50 show thickened septa and fibrotic collapsed alveoli (hyperplasia of type II pneumocytes in detail). Partially obliterated alveoli and alveolar septa of variable thickness are seen in group EOCW100. Wider and more regular alveoli, with thin septa, are observed in groups EOCW200 and DFC (Masson’s trichrome, 400×). (**B**) Assessment of the modified Ashcroft scale of histological gradation of the bleomycin-induced pulmonary fibrosis in the experimental groups. Data are expressed as median, interquartile range, and maximum and minimum values. Significant differences compared to group Vehicle are expressed as ## *p* < 0.01 and ### *p* < 0.001; significant differences compared to group EOCW50 are expressed as ΔΔΔ *p* < 0.001 (Kruskal–Wallis and Dunn’s multiple comparisons test).

BLM induced marked morphologic changes of the lung tissue in the Vehicle group, compared with the Sham group, including: (i) intense inflammatory infiltration, with lymphocytes and alveolar macrophages in the lung interstitium; (ii) severe thickening of the alveolar septa due to intense fibrosis, leading to partial or total obliteration of the pulmonary alveoli (alveolar collapse); (iii) intense peribronchial and peribronchiolar fibrosis; (iv) formation of air bubbles in the middle of the obliterated parenchyma, resembling the honeycomb aspect of hives (alveolar “honeycombing”); and (v) hyperemia and hemorrhage, associated with areas of marked interstitial edema. The treatment with EOCW attenuated BLM-induced lung damage as follows: (i) reduced inflammatory/phagocytic infiltration; (ii) fewer damaged alveoli, including less alveolar obliteration and honeycombing; and (iii) reduced alveolar thickening and peribronchial and peribronchiolar fibrosis. In addition, as observed in DFC (deflazacort-treated group), the pulmonary changes observed in groups EOCW100 and EOCW200 were markedly attenuated to almost normal levels, suggesting that the doses of 100 and 200 mg/kg were more effective in inhibiting PF at a histological level (Figure 3A). As demonstrated in Figure 3B, the severity of the Ashcroft’s histological grading of pulmonary fibrosis induced by bleomycin in the Vehicle-treated group (6; 5−7) was significantly reduced by the use of deflazacort (4, 1−5; *p* < 0.001) and EOCW at 100 (5, 4−6; *p* < 0.01) and 200 mg/kg (5, 4−5; *p* < 0.001) but not with 50 mg/kg (6, 5−6; *p* > 0.05). Furthermore, only the dose of 200 mg/kg of the essential oil promoted histological grading attenuation of the pulmonary damage comparable to deflazacort (*p* > 0.05).

**Figure 4:**
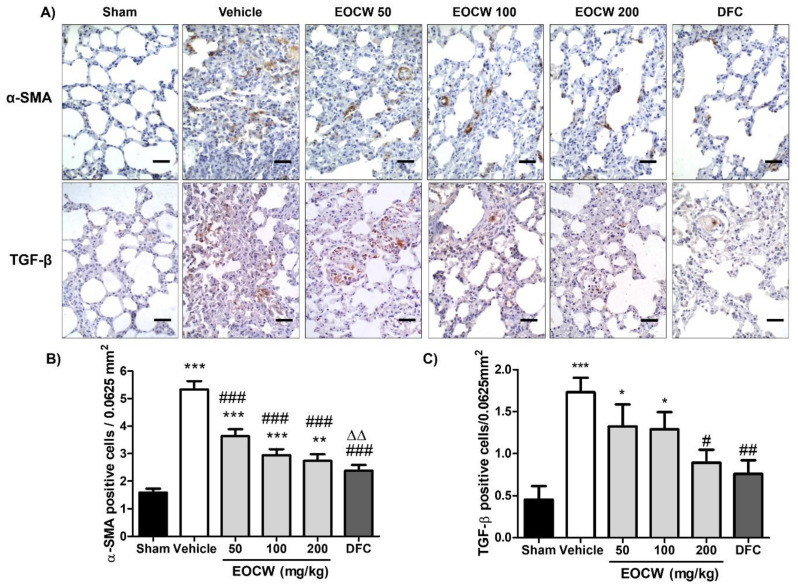
(**A**) Cytoplasmic positivity pattern of immunohistochemical expression of α-SMA and TGF-β in group Sham and subjected to bleomycin-induced pulmonary fibrosis (Masson’s trichrome, 400×). (**B**) Assessment of the immunohistochemical expression of α-SMA and (**C**) TGF-β in the pulmonary tissue of the experimental groups. Significant differences compared to group Sham are expressed as * *p* < 0.05, ** *p* < 0.01 and *** *p* < 0.001; compared to group Vehicle are expressed as # *p* < 0.05, ## *p* < 0.01 and ### *p* < 0.001; compared to EOCW50 are expressed as ΔΔ *p* < 0.01 (ANOVA and Tukey’s multiple comparisons test).

Figure 4 shows the cytoplasmic positivity pattern of the immunohistochemical expression of α-SMA and TGF-β antigens in paraffin-embedded pulmonary tissue of the experimental groups. In group Sham, α-SMA expression in the pulmonary interstitial space and alveolar septa walls was mild (1.6 ± 0.1 cells/0.0625 µm^2^). Although strong positivity was observed in peribronchiolar and perivascular areas, these positive cells were interpreted as smooth muscle cells or eventual pericytes instead of myofibroblasts. No positivity was observed in the alveolar epithelial cells. Group Vehicle exhibited significantly increased α-SMA expression in interstitial cells and type II pneumocytes lining alveolar spaces (5.3 ± 0.3 cells/25 µm^2^, *p* < 0.05). Significant reduction of α-SMA expression was observed in all of the groups undergone treatment with *C. winterianus* essential oil and deflazacort in comparison with the Vehicle group (*p* < 0.001). No significant difference was observed between the groups treated with EOCW 100 (2.9 ± 0.2 cells/0.0625 µm^2^), EOCW 200 (2.7 ± 0.2 cells/0.0625 µm^2^), and DFC (2.3 ± 0.3 cells/25 µm^2^) (*p* > 0.01).

In the Sham group, cytoplasmic immunohistochemical expression of TGF-β was observed in alveolar macrophages, endothelial cells, and interstitial fibroblasts. However, in the groups subjected to bleomycin-induced pulmonary fibrosis, positivity was also seen in bronchiolar epithelium and type I/II pneumocytes. As expected, the immunohistochemical expression of TGF-β in all of the BLM groups was greater than in the Sham group (0.55 ± 0.15 cells/0.0625 µm^2^; *p* < 0.01). The groups Vehicle (3.17 ± 0.21 cells/0.0625 µm^2^) and EOCW 50 (2.661 ± 0.25 cells/0.0625 µm^2^) presented the greatest counts of positive cells, which were both greater than in group EOCW 200 (1.70 ± 0.22 cells/0.0625 µm^2^) (*p* < 0.001 and *p* < 0.05) and DFC (1.40 ± 0.18 cells/0.0625 µm^2^) (*p* < 0.001 and *p* < 0.001). Although the intermediate dose of the essential oil of *C. winterianus* (100 mg/kg) promoted a significant decrease in the immunoexpression of TGF-β (2.44 ± 0.19 cells/0.0625 µm^2^) in comparison with group Vehicle (*p* < 0.05), it was still significantly greater than in 200 mg/kg of the essential oil (*p* < 0.05) and DFC (*p* < 0.001).

## 4. Discussion

Deflazacort is a synthetic glucocorticoid that has few adverse effects on glucose and calcium metabolism, whose pharmacologic safety profile is similar to that of other glucocorticoids. This drug presents strong immunosuppressive and antifibrotic activity [33] and has been used in the treatment of pulmonary fibrosis [34,35]. In addition, the choice for deflazacort in the current study was also based on two other facts: (i) the least adverse effects compared to other drugs; and (ii) its rapid absorption by the gastrointestinal tract when administered orally and immediate hepatic conversion to 21-hydroxideflazacort, its main active metabolite [36].

In this study, the chemical composition of the EOCW was identified by GC-MS and the monoterpenes citronellal, geraniol, and citronellol were the major chemical compounds found. The very same major constituents of EOCW have been previously reported in samples of northeastern Brazil, from Sergipe [37] and Pernambuco [38]. There are some variations in the chemical composition of the oils obtained from plants of the same genus, depending on the soil, location, and seasons when the leaves were collected [39,40,41]. Hence, this could explain other major chemical compositions found in EOCW, as the one reported in the Anand region, northeast India, whose main constituents were citronellol (34.25%), linalool (27.47%), citronellal (11.52%), and emelol (11.15%) [42]. Based on the antioxidant [22,23,24], anti-inflammatory [19,25], and antifibrotic [26] properties of this essential oil, we designed the first study on the potential activity of the EOCW on bleomycin-induced pulmonary fibrosis in a rodent model. In addition, the doses (50, 100, and 200 mg/kg) were chosen according to the study previously conducted by Leite et al. (2011) [19], using EOCW of the same region, with a similar major chemical composition.

As body weight variations are commonly used to monitor an animal’s health status during the progression of diseases, including bleomycin-induced lung injury [43], we assessed the body weight gain in the animals at the end of the time course of the experiment. We found that the administration of bleomycin to induce pulmonary fibrosis promoted loss of body weight in rats over time, which has been also demonstrated in other studies using a similar experimental model [44,45]. Chemotherapeutics, such as bleomycin, causes significant loss of body weight due to the reduction in both muscle mass and as a result of deleterious effects on the gastrointestinal tract [46], which could explain the data obtained in the current study. Only the treatment with EOCW at 200 mg/kg prevented body weight loss, and we hypothesized that such an effect might be related to the high contents of the chemical constituents of the essential oil at this dose. Geraniol administered orally is a modulator of the intestinal microbiota, which is able to improve the relative abundance of *Collinsella* and *Faecalibacterium*, well-known bacteria of health-promoting butyrate [47]. Butyrate is a major metabolite in colonic lumen derived from bacterial fermentation of dietary fiber, responsible for about 70% of energy from the colonocytes [48]. Hence, as butyrate is currently considered a critical mediator of the colonic inflammatory response, it is possible to suppose that the beneficial effects associated with EOCW at 200 mg/kg administration might be related to the high content of geraniol, which was not achieved in lower doses. In addition, the administration of deflazacort provided no significant improvement in the body weight loss. This could be explained by the fact that although the pulmonary inflammatory-induced exudative changes and intense fibrosis can increase the lung weight ~two-fold, it has not been considered enough to significantly mask the animal’s body weight loss [49]. However, further investigations are necessary to fully clarify these theories.

Inhibition of the immune system has been associated with the development of pulmonary fibrosis [50], which led us to assess the hematological parameters of the animals in the current study. Although apparently some significant differences were observed in both red and white cells counts (Table 3), all of the values were within the normal range, and, therefore, they were considered spurious. Similar data have been previously reported [51,52]. It is possible that this result might be related to the experimental model used in the current study, in which the chemotherapeutic agent is applied only once by direct tracheal instillation, producing only mild systemic damage. Hence, these findings are suggestive that the assessment of the peripheral blood red and white cells counts can be unhelpful to evaluate the course of the disease. 

BALF analysis, on the other hand, has shown to determine the severity of alveolitis, and, for this reason, it has been used as a parameter to assess the magnitude of inflammatory damage caused by bleomycin on pulmonary tissues in rodents [53]. Bleomycin intratracheal instillation in group Vehicle determined a significant increase in leukocytes in BALF. Supporting our findings, acute alveolitis and interstitial inflammation, and consequent increased leukocyte recruitment, have been previously reported to occur after intratracheal administration of bleomycin in rodents [54]. Although the exchange between inflammation and fibrosis begins to occur between days 8 and 14, the presence of plasma exudation and, consequently, leukocytes in BALF, between 21 and 28 days has been reported [55,56], as observed in the current study. Hence, these data suggest the long-term persistence of the aggression and the inflammatory response in the lung tissues. In addition, the increase in total proteins has been demonstrated in BALF 28 days after intratracheal bleomycin instillation, which supports the hypothesis of persistent pulmonary exudation [57]. 

A differential analysis of leukocytes in BALF has been used to characterize the nature of the persistent inflammatory response [58]. We found that both polymorphonuclear (neutrophils) and mononuclear cells (lymphocytes and macrophages) increased in BALF, just as previously reported [57]. This leukocyte profile was expected as the BALF analysis was performed on day 28, when the typical acute phase of the inflammatory response had already occurred. Treatment with EOCW reduced the inflammation in BALF in a dose-dependent pattern. In addition, the therapeutic effect of EOCW at 200 mg/kg was statistically similar to deflazacort. These findings suggest that EOCW minimizes bleomycin-induced alveolitis, and that, at a dose of 200 mg/kg, this effect is comparable to the corticotherapy. In a previous study, EOCW at 50, 100, and 200 mg/kg have demonstrated dose-dependent anti-inflammatory properties in rodents [37], which helps to support the hypothesis that the beneficial effects of this natural product might be at least partially associated to the inhibition of the inflammation.

To investigate the role of the antioxidant properties of EOCW in attenuating bleomycin-induced pulmonary fibrosis, we assessed the levels of MDA produced by lipid peroxidation in situ, as well as SOD and CAT activity. We found increased levels of MDA and decreased activity of SOD and CAT in animals subjected to bleomycin-induced pulmonary fibrosis treated with Vehicle only in comparison to Sham, as also observed in previous investigations [59,60]. Bleomycin downregulates phosphorylation expression of mitogen-activated protein kinases (MAPKs), which increases the production of nitric-oxide synthase and NADPH oxidase. The level of MDA, a by-product of lipid peroxidation, is closely related to the oxidative damage of cell membranes that occurs in response to bleomycin-induced lung injury [59]. The tissue activity of the antioxidant enzymes SOD and CAT are some of the most important antioxidant defenses against oxidative stress caused by BLM [61], which could explain the decreased activity of SOD and CAT as observed in the current study. The results presented here suggest that the bleomycin-induced pulmonary fibrosis in the murine model is associated to intense oxidative stress. Hence, oxidative stress is a key pathological process in the development and progression of pulmonary fibrosis, the inhibition of oxidative stress and enhancement of antioxidative ability could supposedly alleviate pulmonary fibrosis [62]. In fact, the treatment with EOCW (100 and 200 mg/kg) reduced MDA levels and increased SOD activity, suggesting antioxidant activity. Supporting our findings, the use of geraniol, one of the major chemical compounds of EOCW, has proved to reduce the lipid peroxidation levels in rat brain tissue homogenates (25–40%) [63]. Geraniol has also been reported to induce activation of nuclear factor erythroid 2-related factor 2 (Nrf2). As upon oxidative stress, Nrf2 detaches from its cytoplasmic inhibitor protein and transfers into the nucleus to activate various antioxidant enzymes (e.g., glutathione peroxidase and superoxide dismutase), the activation of the Nrf2 pathway could be involved in the maintenance of the cellular defense mechanism through antioxidant properties [64]. Furthermore, previous studies have demonstrated that the administration of essential oil of *Rosa damascena* Mill L., whose major constituents are citronellol (38.04%) and geraniol (26.32%), promotes moderate inhibition of MDA production and increase in SOD activity in injured brain tissue [65]. So, although the precise mechanisms underlying the antioxidant activity of EOCW are not fully clarified, we hypothesized that the EOCW ability to inhibit or reduce the oxidative stress and inflammatory response could be involved in the attenuation of the tissue damage that occurs in bleomycin-induced pulmonary fibrosis.

The occurrence of severe pathological alterations in the lung tissue in response to bleomycin intratracheal instillation is well established [66], and, for this reason, we investigated the effects of EOCW on the prevention or attenuation of the histological changes of the injured lungs. Hyperplasia of type II pneumocytes, massive infiltration of alveolar macrophages, and thickening of the alveolar septa, leading to total or partial obliteration of the alveoli, were some of the morphological features found in the bleomycin-injured lungs, which are in accordance with other studies [60,67]. As the modified Ashcroft scale has been widely used to grade the severity of the histological damage associated to bleomycin-induced pulmonary fibrosis [31], it was applied in the current study. Attenuation of the pulmonary histological damage resulting from the administration of EOCW at doses of 100 and 200 mg/kg was found in the current study, which might be possibly related to the anti-inflammatory and antioxidant properties of the major chemical compounds of the essential oil [68,69,70]. In fact, citronellal has shown to inhibit 5-lipoxygenase and nitric oxide production [23], whereas citronellol inhibits COX-2 and prostaglandin-E2 (PGE2) expression, and impairs TNF-α-induced neutrophil adhesion, as well as increases SOD production, reduces NO release, and decreases inducible nitric oxide synthase activity [24]. Furthermore, geraniol inhibits prostaglandin E2 and tumor necrosis factor alpha (TNF–α) [71], and has been considered a potential drug to be used in inflammatory lung diseases, where oxidative stress was a critical point [22]. Therefore, the control of the inflammatory response and oxidative stress will be instrumental to prevent or attenuate the progression of the histological damages that features the pulmonary fibrosis. Supporting this theory, the progression of the histological damage was also achieved using corticosteroids (deflazacort). At 50 mg/kg, the EOCW was unsuccessful in preventing BLM-induced pulmonary fibrosis, supposedly due to the low concentrations of the chemical compounds at this dose. In addition, although the results obtained with 100 mg/kg were significantly better than using the Vehicle only, they were not comparable with deflazacort. Taken together, these data pointed at a dose-dependent effect played by EOCW on the prevention of the pulmonary fibrosis induced by BLM.

There are significant parallels between inflammatory response and myofibroblast differentiation in fibrosis of different tissues [72], and myofibroblasts play a key role in fibrogenesis via the accumulation of an excessive amount of extracellular matrix in lungs with idiopathic PF [14,73]. Hence, the development of effective therapeutic interventions against idiopathic PF have focused on the reduction or prevention of myofibroblast over-differentiation [74]. As in histological sections, myofibroblasts can be easily marked and quantified by the immunohistochemical detection of cytoplasmic filaments of α-smooth muscle actin (α-SMA) [75]; we assessed the pulmonary immunohistochemical expression of α-SMA-positive cells in the current study. Increased counts of α-SMA positive myofibroblasts were found in animals with bleomycin-induced pulmonary fibrosis, which is in accordance with previous studies using the same experimental model [54,76]. These data suggest that the pathogenesis of pulmonary fibrosis can be associated with increased recruitment of myofibroblasts.

Although the precise origin of myofibroblasts in pulmonary fibrosis is controversial, studies have suggested that they likely derive from TGF-β activated preexisting peribronchial and perivascular adventitial fibroblasts and pericytes, migration of smooth muscle cells from adjacent areas, as well as EMT of type II pneumocytes and endothelial cells [77,78]. As a response of EMT, these immotile epithelial cells are converted into motile mesenchymal cells with a myofibroblastic phenotype, characterized by the loss of expression of typical epithelial markers (e.g., E-cadherin) and increased expression of mesenchymal and contractile markers (e.g., vimentin and α-smooth muscle actin, respectively) [79]. There is recent evidence that EMT-derived myofibroblasts present higher rates of proliferation and collagen production than conventional fibroblasts, and, therefore, they play an important role in the pathogenesis of idiopathic PF [10]. The major cytokine involved in the myofibroblast differentiation, irrespective to its origin (mesenchymal or epithelial), is TGF-β released by T cells, macrophages, activated endothelial cells and smooth muscle cells, and fibroblasts under stress conditions [80]. In addition, enhanced in situ release of TGF-β1 promotes deregulation of the Wnt-β-catenin signaling pathways, which confers resistance to apoptosis and proliferative advantages to myofibroblasts. Thus, an imbalance between profibrotic and antifibrotic mediators is created, maintaining an environment supportive of exaggerated myofibroblast activity and chronic fibroproliferation [2].

Myofibroblast differentiation was reduced in response to the treatment with EOCW and deflazacort. The precise mechanisms underlying the negative modulation of myofibroblast differentiation by EOCW is not fully clear. Inhibition of in vitro and in vivo myofibroblastic differentiation potential through blockage of TGF-β1 expression exerted by corticosteroids in palmar fibromatosis-derived stem cells has been previously reported [81]. As the corticoid used in the current study also reduced myofibroblastic differentiation, this inhibitory activity might have been a result of TGF-β1 suppression. Inflammatory cells and other epithelial and mesenchymal cells activated by inflammatory cytokines are the major sources of TGF-β. Therefore, drugs such as steroids and cyclooxygenase inhibitors have been considered effective in reducing inflammatory cytokines, such as TGF-β, and consequently attenuate collagen deposition [82].

As far as we know, no previous study has focused on the inhibitory effects of the major chemical compounds of EOCW on myofibroblastic differentiation, but we hypothesized that it might be related to their anti-inflammatory activity and suppression of TGF-β expression—just as demonstrated with corticosteroids. To prove this theory right, the immunohistochemical expression of TGF-β was assessed in histological sections of lungs with BLM-induced pulmonary fibrosis. As expected, untreated animals presented the greatest counts of TGF-β-expressing cells, which agrees with other studies previously reported [56,82,83]. The significant decrease in pulmonary cells expressing TGF-β in EOCW-treated groups at 100 and 200 mg/kg, as well as in the deflazacort-treated group, is fully supported by the analysis of α-SMA-positive cells, attesting to the close relation between this cytokine and myofibroblast differentiation. In addition, as also observed in the current study, other investigations have demonstrated the attenuation of the severity of the pulmonary fibrosis through the use of drugs that suppress TGF-β expression, such as halofuginone, crocin [79], and amitriptyline [9]. Oral administration of angiotensin 1–7, a heptapeptide with anti-inflammatory activity, in a model of BLM-induced lung fibrosis in mice, decreased inflammation and collagen deposition, as well as ameliorated lung function [83], whereas the incubation of human lung fibroblasts with A779, an angiotensin 1–7 agonist, has shown to reduce levels of TGF-β and collagen type [84]. These data seem to support the relation between anti-inflammatory agents and downregulation of TGF-β, with consequent reduction of fibrosis.

## 5. Conclusions

Oral administration of EOCW attenuates the histological changes associated to the progression and severity of bleomycin-induced pulmonary fibrosis in rodents, likely due to inhibition of TGF-β immunohistochemical expression and the consequent decrease in the myofibroblast differentiation. We also provide evidence that this antifibrotic activity could be associated with the anti-inflammatory and antioxidant properties of the essential oil. Therefore, EOCW is a potential candidate to be used as a phytotherapeutic in further clinical trials for the treatment of pulmonary fibrosis.

## Figures and Tables

**Table 1:** Distribution of animals in experimental groups according to treatment.

Groups (*n* = 6)	Pulmonary Damage Procedure	Oral Administration (Treatment) ^a^
Sham ^b^	Saline solution	Soybean oil
Vehicle ^c^	Bleomycin sulfate (5 mg/kg)	Soybean oil
EOCW 50 ^c^	Bleomycin sulfate (5 mg/kg)	50 mg/kg essential oil of *Cymbopogon winterianus*
EOCW 100 ^c^	Bleomycin sulfate (5 mg/kg)	100 mg/kg essential oil of *Cymbopogon winterianus*
EOCW 200 ^c^	Bleomycin sulfate (5 mg/kg)	200 mg/kg essential oil of *Cymbopogon winterianus*
DFC ^c^	Bleomycin sulfate (5 mg/kg)	1.0 mg/kg Deflazacort

^a^ The volume administered was 0.2 mL, regardless of the treatment. ^b^ Intratracheal instillation of saline and ^c^ bleomycin to induce pulmonary fibrosis.

**Table 2:** Modified Ashcroft scale for histological grading of lung damage.

Grade of Fibrosis	Modified Scale
0	Alveolar Septa: No fibrotic burden at the flimsiest small fibers in some alveolar walls Lung structure: Normal lung
1	Alveolar Septa: Isolated gentle fibrotic changes (septum ≤3× thicker than normal) Lung structure: Alveoli partly enlarged and rarefied, but no fibrotic masses present.
2	Alveolar Septa: Clearly fibrotic changes (septum >3× thicker than normal) with not-like formation but not connected to each other Lung structure: Alveoli partly enlarged and rarefied, but no fibrotic masses.
3	Alveolar Septa: Contiguous fibrotic walls (septum >3× thicker than normal) predominantly in whole microscopic field Lung structure: Alveoli partly enlarged and rarefied, but no fibrotic masses.
4	Alveolar Septa: Variable Lung structure: Single fibrotic masses (≤10% microscopic field)
5	Alveolar Septa: Variable Lung structure: Confluent fibrotic masses (>10% and ≤50% of microscopic field). Lung structure severely damaged but still preserved.
6	Alveolar Septa: Variable, most not existent Lung structure: Large contiguous fibrotic masses (>50% of microscopic field). Lung architecture mostly not preserved.
7	Alveolar Septa: Non-existent Lung structure: Alveoli nearly obliterated with fibrous masses but still up to five air bubbles.
8	Alveolar Septa: Non-existent Lung structure: Microscopic field with complete obliteration with fibrotic masses.

**Table 3:** Chemical composition and retention indices of the chemical constituents of the EOCW.

RT (min) ^a^	Compounds ^b^	(%) ^c^	RI ^d^
6.4	3-Hexanone, 2-methyl-	0.01	871
11.0	5-Hepten-2-one, 6-methyl-	0.03	987
11.1	β-Myrcene	0.10	990
12.7	Limonene	3.21	1027
13.9	Melonal	0.16	1053
15.4	Terpinolene	0.05	1087
16.0	Linalool	1.17	1101
16.5	*cis*-Rose oxide	0.05	1110
17.2	*trans*-Rose oxide	0.03	1127
18.1	Isopulegol	1.55	1145
18.7	Citronellal	32.61	1159
19.2	Isopulegol	0.10	1170
19.6	4-Terpineol	0.07	1178
19.8	Carane, 4,5-epoxy-, *trans*	0.06	1183
20.2	α-Terpineol	0.09	1191
20.3	*cis*-4-Decenal	0.05	1194
20.8	Decanal	0.20	1206
22.1	Citronellol	14.37	1233
22.5	Neral	1.32	1242
23.4	Geraniol	22.83	1262
23.8	Geranial	1.56	1272
25.9	Citronellic acid	0.26	1320
27.4	Citronellol acetate	0.93	1354
27.6	Phenol, 4-allyl-2-methoxy-	0.94	1359
28.7	Geranyl acetate	1.14	1384
29.0	β-Element	0.56	1392
30.1	Caryophyllene	0.04	1419
31.5	Humulene	0.07	1454
32.5	Naphthalene, 1,2,4a,5,6,8a-hexahydro-4,7-dimethyl-1-(1-methylethyl)-	0.07	1477
32.7	Germacrene D	0.97	1482
33.4	α-Muurolene	0.30	1501
33.6	δ-Guaiene	0.56	1506
34.0	α-Amorphene	0.24	1514
34.4	β-Cadinene	1.16	1524
35.4	*o*-Menth-8-ene-4-methanol, α,α-dimethyl-1-vinyl-, (1S,2S,4R)-(−)-	3.64	1552
36.4	*trans*-Sesquisabinene hydrate	1.02	1577
36.7	Caryophyllene oxide	0.14	1584
38.1	Eudesmol	0.49	1621
38.4	α-Acorenol	0.46	1629
38.5	α-Eudesmol	1.26	1633
38.9	α-Muurolol	1.79	1644
39.0	Cadinol	0.45	1648
39.2	β-Eudesmol	0.67	1652
39.4	α-Cadinol	2.95	1657
41.5	(Z,E)-Farnesol	0.04	1716
41.7	(E,E)-Farnesol	0.21	1723

^a^ RT, retention time; ^b^ compounds listed in order of elution from an DB-5MS column; ^c^ percentage based on FID peak area normalization; ^d^ RI, retention index, calculated using the Van den Dool and Kratz (1963) equation [29].

**Table 4:** Body weight variation of animals according to the experimental group.

Body Weight	Sham	Vehicle	EOCW 50	EOCW 100	EOCW 200	DFC
Initial (g)	174 ± 11.33	236.8 ± 20.7	213.33 ± 20.81	200.25 ± 12.01	179.83 ± 9.04	174.66 ± 6.31
Final (g)	183.4 ± 9.55	228.6 ± 15.14	206.66 ± 20.64	195 ± 7.02	182.5 ± 8.31	172.5 ± 6.22
Body weight gain (%)	5.17 ± 1.60 ^a^	−3.46 ± 2.32 ^b^	–3.24 ± 1.21 ^b^	–2.63 ± 2.85 ^b^	1.42 ± 0.55 ^c^	–1.27± 0.48 ^b,c^

Data are expressed as means ± SEM. Different letters (a, b, c) in the same line represent significantly different values between them (*p* < 0.05; ANOVA and Tukey’s multiple comparisons test).

**Table 5:** Hematological parameters of the animals submitted to the experiment.

Hematological Parameters	Sham	Vehicle	EOCW (mg/kg)			DFC	Reference Range *
			50	100	200		
Total leukocytes (×10^3^/µL)	4.7 ± 0.6 ^a^	3.1 ± 1.2 ^b^	5.0 ± 0.3 ^a^	4.5 ± 0.6 ^a^	6.1 ± 1.4 ^a^	3.6 ± 0.4 ^b^	2.3−9.9
Red blood cells (×10^3^/µL)	8.0 ± 1.0 ^a^	5.8 ± 0.7 ^b^	6.1 ± 1.7 ^b^	6.1 ± 0.9 ^b^	7.6 ± 1.2 ^a^	9.0 ± 0.4 ^a^	5.2−8.8
Hematocrit (%)	51.8 ±1.9 ^a^	38.4 ± 4.0 ^b^	40.5 ± 0.70 ^b^	39.2 ± 1.70 ^b^	46.0 ± 2.4 ^a^	52.8 ± 2.9 ^a^	27.2–48.5
Hemoglobin (g/dL)	15.7 ± 0.9	13.7 ± 1.7	13.6 ± 0.9	13. 9 ± 0.8	14.6 ± 1.0	16.0 ± 0.8	11.1–17.1
Platelets (×10^3^/µL)	1.0 ± 0.2	1.3 ± 0.7	1.0 ± 0.4	1.2 ± 0.2	0.9 ± 0.1	0.8 ± 0.1	0.76–1.31

* Reference range values available in Lima et al. (2014) [32]. Different letters (a, b) in the same line express significantly different values between them (ANOVA and multiple comparison Tukey’s test, *p* < 0.05).

## Data Availability

Data available from authors upon request.
